# Consolidating Interprofessional Learning Using an Escape Room in a Briefcase Activity in a Rural Setting

**DOI:** 10.1111/ajr.70196

**Published:** 2026-04-20

**Authors:** Kate Beyer, Lucy Parker, Christine O'Connell, Dayle Osborn, Kirsten Middleton, Emma Gordon, Flora Rolf, Anthony Fallon

**Affiliations:** ^1^ Australian Catholic University Brisbane Australia; ^2^ Southern Queensland Rural Health University of Queensland Toowoomba Australia

## Abstract

**Objective:**

The Escape Room in a Briefcase (ERiB) is a highly portable activity for students to observe and practice interprofessional practice competencies. The ERiB was incorporated into a University Department of Rural Health's Student Workshop in Interprofessional Education (SWIPE) to facilitate delivery of effective interprofessional education (IPE) to students on placement in rural and regional locations. The evaluation aimed to understand the students' gaming experience and improvement in understanding of interprofessional competencies and to gain feedback about the ERiB activity.

**Setting:**

Health facility‐based education rooms across six different locations in the Darling Downs and Southwest Queensland.

**Participants:**

Eighty‐seven university students who were undertaking a clinical placement for a health degree in the Darling Downs or Southwest Queensland participated in one of 13 SWIPE sessions over a 2‐year period.

**Design:**

The research utilised an interpretivist approach to qualitative design, with data collected via semi‐structured focus groups with students after the ERiB activity.

**Results:**

Analysis revealed four key themes: frustration during teamwork, collaborative capability, situational humility and intrinsic motivation, which were interpreted as core capabilities underpinning interprofessional practice.

**Conclusion:**

The ERiB activity is an exciting and innovative method that provides IPE opportunities for students on placement in any location. This interactive activity appears to succeed as an effective IPE tool, facilitating skills in communication, teamwork and collaboration.

## Summary

**What is already known on this subject:**

- Interprofessional education is a critical component of high‐quality student placements.
- Working in rural health requires unique skills.
- Gaming in learning is an effective tool to teach interprofessional practice.
- Good facilitation and debriefing are essential elements of learning activities.

**What this paper adds:**

- Demonstrates a feasible gaming activity that is not reliant on software, is highly portable, and enjoyable for players.
- Provides evidence that this is an effective tool that:
  ○ Meets different learning styles of students, enabling all learners to participate thereby enhancing collective problem solving and strengthening professional identity.
  ○ Levels the ‘playing field’ in interprofessional collaboration by eliminating perceived professional hierarchies, fostering situational humility.
  ○ Is reflective of rural healthcare and its workforce, being complex, dynamic and requiring ‘teaming’ skills for effective collaboration to achieve shared goals.

## 1 Introduction

Interprofessional collaborative practice has been identified by the World Health Organisation (WHO) as an innovative strategy to improve health outcomes and strengthen health systems. Interprofessional education (IPE) occurs when students from two or more professions ‘learn about, from and with each other’ [1]. Effective IPE is required to ensure health students are job ready and prepared to work collaboratively in an interprofessional team. IPE provides collaborative teamwork experiences that promote understanding of professional roles and professional autonomy whilst also providing essential knowledge and skills for improving healthcare services. Healthcare has become increasingly complex, with the composition of interprofessional healthcare teams changing frequently, and health professionals needing to adapt to interprofessional collaborative practice within this shifting workforce environment [2]. Interprofessional collaboration is often applied ‘on the fly’ to a clinical situation which can be novel in nature. This concept, referred to as teaming [3], is representative of the reality of many interprofessional healthcare encounters.

An innovative strategy for enhancing IPE is the use of game‐based learning, which combines educational objectives with interactive game elements to create engaging and impactful learning experiences. This approach has been found to increase student motivation, engagement and knowledge retention by offering a dynamic, immersive environment [4, 5]. In IPE, game‐based activities can replicate real‐world challenges that require effective communication, teamwork and problem‐solving. An added benefit of escape room‐type games is their ability to level the playing field by minimising traditional hierarchical roles often present in healthcare settings [6]. This allows students to safely develop and refine these essential skills in a simulated environment, better preparing them for real clinical practice.

An important focus for one University Department of Rural Health (UDRH), based in southern Queensland, is to promote interprofessional collaborative practice through the provision of IPE to health students, with the long‐term goal of cultivating a sustainable, collaborative health workforce in rural and remote locations.

To enhance student learning when on placement in rural and remote settings, the UDRH provides a range of IPE opportunities. The UDRH has collaborated with a local company to design several portable escape rooms, which are completely enclosed within a briefcase to enable transportation. The Escape Room in a Briefcase (ERiB) activity consists of a series of puzzles scaffolded by a clinical scenario but not requiring clinical skills to be solved. The puzzles need to be solved sequentially to ‘escape’. ERiB is integrated into the UDRH Student Workshop in Interprofessional Education (SWiPE), with the goal of providing a portable activity where complex game‐based problems can be used to elicit and discuss interprofessional learning and teaming concepts.

This research aimed to investigate the impact of using the ERiB as an innovative, fun way to consolidate a health student's interprofessional learning and to facilitate the use of teaming skills within the context of an IPE workshop.

## 2 Methodology

An interpretivist approach was adopted for this research as it sought to explore students' subjective perceptions of the Escape Room activity, including how they experienced IPE, teamwork and engagement during the activity. An interpretivist theoretical framework recognises that reality is socially constructed and that meaning is developed through individual experiences and interactions [7]. Rather than measuring predetermined outcomes, the study aimed to understand how students interpreted and made meaning of their learning experiences within a shared educational context. The research team used focus groups with students to gain an understanding of whether ERiB was a fun, positive learning activity that effectively consolidated IPE learning and teaming concepts.

### 2.1 Setting

The UDRH is funded through the Rural Health Multidisciplinary Training program and provides support and training opportunities to allied health, nursing and midwifery students on placement across the Darling Downs and Southwest region of Queensland. IPE opportunities were conducted with students across the UDRH footprint.

### 2.2 Participants

Participants were health students on placement in the Darling Downs and Southwest regions of Queensland from a variety of Australian Universities. Students were eligible for inclusion when they presented in person to undertake the SWIPE with integrated ERiB whilst on their clinical placement. Students were invited to the workshops by their clinical facilitators, via social media, email, or the UDRH website. They self‐registered for attendance via the URDH events webpage. There was no prior relationship between the students and research team members conducting the interviews.

Students were made aware that the ERiB activity within SWIPE was part of a research study and were invited to participate. Students were also able to take part in SWIPE and ERiB without participation in the study, though no students opted not to participate.

### 2.3 ERiB Design

The ERiB is structured around a clinical scenario within which a series of puzzles are embedded. Students are briefed prior to commencing: they have 1 h to complete the activity, must not use force or tamper with locks, and are encouraged to be curious, work as a team, and enjoy the process. Each puzzle involves completing a task using an object or tool to generate a code, which unlocks the next stage and reveals further elements of the patient or family story. The final puzzle requires students to determine the most appropriate interprofessional management for the case.

The ERiB is designed to be portable, weighing under 7 kg, allowing easy transport by car or as carry‐on luggage, and use in small spaces. Puzzles are scaffolded by clinical scenarios developed by the research team. These reflect rural healthcare challenges, such as distance and limited access to services, without requiring specific clinical knowledge (see Appendix A). This allows participation across all health disciplines and levels. Students work in groups of 4–6 per briefcase.

Facilitators support progression to ensure timely completion, providing hints and prompts to encourage collaboration and engagement where needed.

### 2.4 Data Generation

After students provided their informed consent, they engaged in the SWIPE which included the ERiB as an integrated activity. Immediately following the ERiB, students completed a debrief about their experiences. This also served as an opportunity for IPE facilitators to reinforce the relationship between IPE competencies and interactions during the ERiB activity. Students were asked to identify instances in which interprofessional education (IPE) competencies were demonstrated within the activity. The facilitators provided their own examples from the activity to demonstrate connection to IPE competencies. The debrief also allowed facilitators to highlight challenges present in the scenario and how interprofessional practice impacted the outcome for the patient/family in the scenario. This opportunity for consolidation of learning was imperative to integrate the ERiB activity and its overall relationship to the IPE experience.

Immediately after the debrief, student willingness to participate in the study was confirmed and participation in the focus groups occurred. The interviews occurred in the room in which the workshop took place or a nearby room. All rooms were designated education rooms on site at the placement facility or the UDRH.

Semi‐structured focus groups were facilitated by the UDRH Clinical Educators who were also members of the research team and had combined clinical, research and education experience. The facilitators differed based on the workshop location, aligning with the Clinical Educator's usual workplace. An interview guide consisting of broad, open‐ended questions was used to explore students' experiences of the ERiB, including its role in supporting understanding of IPE competencies, the gaming experience, and its utility in fostering teaming skills (see Appendix B). The guide was pilot tested and was consistent with an inductive approach. Interviews remained flexible, allowing for probing and follow‐up questions. Two researchers were present at each focus group, one facilitating and one taking notes. Data generation included audio recordings, field notes and collated group summaries. Each focus group lasted approximately 45 min. The project was conducted over a 2‐year period. Data saturation was discussed toward the end of the project; however, data generation ceased due to the project timeframe and the conclusion of scheduled SWIPE and ERiB activities.

## 3 Findings and Analysis

Consistent with an interpretivist methodology, analysis focused on understanding how students interpreted their experiences of the ERiB activity, how meaning was constructed through social interaction, and how shared reflection influenced their understanding of interprofessional learning and teamwork. Students' accounts illustrate that learning was not experienced as a fixed outcome of the activity, but rather as something actively negotiated through emotion, dialogue, collaboration and reflection.

An inductive thematic analysis was undertaken following Braun and Clarke's [8] approach. This allowed for flexibility and is suitably aligned with an inductive analysis. This method is useful when exploring shared meanings, group dynamics and patterns across a participant group [8]. Audio‐recorded focus groups were transcribed verbatim using the Microsoft Word transcription function and checked for accuracy by the focus group facilitators. Relevant quotes were identified, time‐tagged and transcribed for use as verbatim support for identified themes. Initial codes were generated inductively and progressively refined through ongoing comparison, discussion and clustering into conceptual themes. Relevant literature on IPE, gaming in health education, and the impact of teaching and learning on student and professional identity informed this analytic process. Both semantic content and latent meanings were examined, with particular attention given to the affective dimensions of students' learning experiences.

Four research team meetings were held to review, define and name themes, with consensus reached regarding thematic relationships and alignment with relevant theoretical frameworks. Conceptual insights derived from the themes informed interpretation of student perceptions. Quotations were de‐identified and assigned codes. Participants were not provided with transcripts or invited to review findings. Although participant validation can enhance credibility in some qualitative designs, it was not considered appropriate in this study. As data were generated through focus groups, where contributions are co‐constructed and shaped by group dynamics, returning transcripts risked compromising participant confidentiality. The study also adopted an approach in which meaning was understood as contextually situated and analytically constructed through researcher reflexivity rather than requiring verification. The analytic focus was on shared patterns and thematic interpretation rather than individual narratives. Trustworthiness was therefore established through team‐based coding, reflexive discussion, audit processes and transparent documentation of analytic decisions.

Data were generated from 87 students across 13 SWIPE sessions between October 2021 and October 2023. The students ranged between 19 and 58 years with a median age of 24. Seventy‐two (82.8%) were female, with the remainder identifying as male. Placement length was reported by 84 (96.6%) students, and the length of placement ranged between 2 and 21 weeks, with a mean placement length of 7.6 weeks (SD = 3.9 weeks). During their participation, students were completing clinical placements in regional, rural and remote towns across Southern Queensland. According to the 2019 Modified Monash Model (MMM), which classifies areas from MM1 (metropolitan) to MM7 (very remote), these sites fell within MM2–MM7 and included Toowoomba, Chinchilla, Roma, Charleville, Kingaroy and St George [9].

If the number of students in the SWIPE workshop was greater than six, two ERiB activities were run concurrently. Overall, a total of 20 ERiB activities occurred, and 20 focus groups were conducted.

The focus groups revealed four overarching themes:

- Theme 1—Frustration during teamwork.
- Theme 2—Collaborative capability.
- Theme 3—Situational humility.
- Theme 4—Shared goal as an intrinsic motivator.

### 3.1 Theme 1: Frustration During Teamwork

Yeah, I think that's like obviously stress like frustration stress. But then also like motivational stress, like when you get a little bit stressed out, you think harder you, umm… Like, yeah, start thinking outside the box. SP4/ER22021

The recollection of frustration was a commonly expressed notion among students in focus groups. Yet, frustration was repeatedly discussed as a developmental or even integral part of team formation and goal realisation. Participants often supported an expression of frustration with qualifiers around the benefit of getting frustrated, or a measured acceptance of frustration as part of a broader team process.

Students described some of the inherent challenges associated with teamwork without dismissing the value, and many were able to identify their own responses to teamwork rather than externalising them. Exploring these recollections as affective or emotional responses to learning and the integration of new knowledge offered some insight into the group's shared meaning‐making of the role of emotion.

Lodge et al. [10] centre the importance of in‐person and group learning in this space, arguing that detecting and adjusting group actions according to others' emotions is a key reward of in‐person activity. The work of students during ERiB suggested a broad appreciation of affective ‘prickles’ and the prompt to then turn to cognition and knowledge formation to work through frustration, confusion and reach shared goals:

I think some people like probably prefer to work alone and like it can, it can be frustrating like working in a team like trying to problem solve because you all have like different thinking styles and that element can be frustrating. SP3/ER22023

…Oh, more just frustration. Not at any person in particular, but the situation…. SP4/ER12022

The concept of frustration in learning draws on behavioural psychology research [11] and the concepts of persistence and discernment in learning. More recently D'Mello et al. [12] and Davison et al. [13] conflate confusion and frustration, proposing a positive pedagogical outcome. Confusion and frustration are often labelled as affective, yet D'Mello et al. [12] argue it also serves an epistemic and knowledge‐based function by requiring, applied to this context, a group of students to assess new and untested information against their own assumptions and experiences about working as a team and achieving shared goals.

The collective sense‐making that frustration was a necessary part of their quest to realise the activity goals together shaped the work of the students, generating a transformative learning experience. The students used affective and cognitive knowledge integration as a catalyst to develop their team and reach a set of shared goals, indicating a convincing association with interprofessional practice principles:

Frustrated <laughter from the group>, at first. Yeah, but then it was nice, yeah, feeling part of a team and getting stuff done together, it was good, yeah. SP1/ER12021

### 3.2 Theme 2: Collaborative Capability

Students identified a key strength of the ERiB activity was the opportunity to collaborate with peers who demonstrated diverse learning styles, communication preferences and personality traits. Working within heterogeneous teams enabled multiple perspectives, thereby enhancing collective problem‐solving:

each person has their own like unique set of skills and excel in a certain environment that you might not, and then vice versa, they may need help in an environment that you have more knowledge in or understand better and then that can be a working relationship between you and another or you and the whole team and you figure out what your skills are where you need improvements. SP5/ER32011

Students demonstrated insight not only into their own learning preferences but also into those of their peers. Despite generational and interpersonal differences, participants described valuing and actively encouraging contributions from all team members. This attentiveness to others' needs, alongside recognition of their own, appeared to foster a collegial and inclusive orientation distinct from traditional classrooms [14, 15]:

I think it gives everyone that opportunity to shine with their own skills, because there's enough different aspects of it that it all comes together and it allows everyone to actually be part of it. And you know, part of that actual team with a good contribution. SP3/ER32011.

The collaborative and mutually accountable nature of this team‐based approach is consistent with Self‐Determination Theory (SDT). Within SDT, relatedness—one of the three core psychological needs—reflects the fundamental desire for meaningful connection and belonging [16]. Students' accounts underscored the importance of a shared goal in fostering cohesion:

I think because we all had a common goal and I guess that's important too when everybody wants exactly the same outcome it makes it a little bit easier. If we were trying to do different things or if we were trying to do it for our own purposes it might have been different. But we all wanted to open the cases. SP4/ER12022.

Supporting others out of genuine connection rather than obligation reflects altruistic and prosocial behaviours [16, 17]. Development of collaborative capability may therefore underpin empathy, perspective‐taking and cooperative engagement, all essential in clinical contexts, and contribute to professional identity formation [18].

Students further described recognising their own limitations and valuing the strengths of others as integral to both puzzle completion and future professional practice:

it was clear that everyone had different unique contributions and different ways of thinking about things, and of course the psychological safety of being able to voice our opinions or thoughts. And I think that also speaks to being humble, realising that your idea might not be the be all and end all but yeah, so you need to listen to your team. SP6/ER22021

### 3.3 Theme 3: Situational Humility

Students articulated the concept of situational humility, describing awareness of professional hierarchies, acknowledgement of personal limitations, and appreciation of others' capabilities. Expressions of openness and acceptance appeared to foster psychological safety within teams:

I think having to compensate everyone's, needs and roles as the situation changes kind of thing. Like, in a certain situation, like at the start someone might be able to take a bit of charge, but then the situation might change and then someone else needs to take charge because they maybe have a little bit more knowledge around that sort of thing so that's I think how it would work there. SP2/ER32023

All of us together we were able to keep coming up with ideas of things to try and then eventually we found the right ones. You know, no one of us has the creativity of the six of us combined to think of all of them so that was really cool. SP1/ER32023

Humility within the team context could also be seen in the SDT's core psychological needs. Self‐assessment, openness to new ideas and valuing others' contributions could be argued to enhance perceived competence and relatedness [19]. Although demonstrating humility across professional groups can be challenging [20], ERiB provided a de‐siloed learning environment in which previous professional hierarchies were minimised. The activities within ERiB require no clinical skills and being new to all students allowed for a ‘level playing field’:

And also that it's outside of anyone's discipline. So, you're coming into it as people without titles or hierarchy or anything like that… SP5/ER32023

This ‘level playing field’ facilitated collaborative practice and reduced perceived status differentials, consistent with literature emphasising humility as foundational to interprofessional practice [21]:

But in this situation we all felt like we're on the same page, we all have different skills, we're working together to try and solve the escape room. So yeah, it was that, that collaborative practice I guess, yeah, coming together. But not feeling like anyone was better than anyone else. And it just made it more easier to open up, I guess. SP1/ER32023

### 3.4 Theme 4: Intrinsic Motivation

In addition to themes of frustration, collaborative capability and situational humility, students described feelings of positive stress and interest in working together as ‘motivating’ factors to continue to progress through the varying ERiB puzzles:

But then also like motivational stress, like when you get a little bit stressed out, you think harder you, umm… Like, yeah, start thinking outside the box. SP2/ER22021

just being with the team and having different ideas … push you along and to progress to that common goal. SP4/ER12021

Factors encouraging people to ‘push on’ and ‘keep going’ are considered as causal forces acting on individuals rather than inherent behaviours. Motivation then is seen as a psychological force, arising from within or from external influences, energising behaviour and continued effort toward a goal. Within SDT, intrinsic motivation is grounded in the needs for autonomy, competence and relatedness [16]. ‘Autonomy’ is seen as an individual's behaviour regulated by the self but not excluding dependence on others, with the focus being on the individual's volition and a sense of integrity and alignment with values [22]. Students described enjoyment, engagement and satisfaction when progressing collaboratively:

it was very interactive and we got to communicate and work as a team as well as we got something right or when we're headed in a way that felt right, it was more satisfying as well. So kept us engaged and the whole thing. SP1/ER32023

Students also reported competence and confidence, despite feeling challenged at times:

there were parts that challenged us but we didn't throw in the towel. SP5/ER12023

Within SDT, the psychological need of ‘competence’ refers to the experience of efficacy and growth coming from interaction with the environment/activities, an appropriate level of challenge leading to an extension of abilities and mastery [16]. Elements supporting competence include encouraging reflection, timely and constructive feedback and managing complexity [23].

Intrinsic motivation was reflected in experiences of social connectedness and collaborative engagement. Students drew encouragement from within the interprofessional team, reinforcing collective effort toward a shared goal:

with the scenario you have no idea and you have to work between yourselves to actually have the end goal but work together, collaboratively, to make it happen. SP3/ER12022.

## 4 Discussion

With the increasing complexity of healthcare delivery, particularly in rural and remote settings, there is a need for innovative educational strategies that foster collaboration, adaptability and strong interprofessional competencies among health students. When considering the current demographics of health students, traditional teaching models often fall short in addressing the diverse learning preferences and professional hierarchies that can inhibit effective team‐based learning [6]. Furthermore, unlike many existing escape room activities, ERiB was developed with rural portability as a core requirement. Logistical barriers associated with transporting and setting up escape rooms often require dedicated space and resources that may not be available in rural areas [24]. This project demonstrates that a portable, game‐based learning activity can be a highly effective tool for enhancing IPE outcomes in rural placements through the lens of situational humility, reflection, persistence, adaptability and collaborative team qualities.

The feeling of being ‘stuck’ and frustrated can lead to negative experiences and avoidance which can be perceived as detrimental to learning [10]. However, confusion and frustration can also play a role in reflection and learning [10]. Students' reflections during the ERiB activity suggest that frustration functioned not as a barrier to learning, but as a productive and shared affective experience that stimulated persistence, reflection and collective problem‐solving. The integration of emotional and cognitive processes enabled teams to reinterpret confusion as part of developmental growth. Whilst negative emotions such as frustration are inevitable and can be paralysing, health professionals who are able to reflect and learn from the experience can influence the effectiveness of interprofessional teamwork [25]. This reframing of frustration appeared to support transformative learning and alignment with interprofessional practice principles.

Tacit team qualities such as adaptability, responsiveness and openness are integral to interprofessional practice [26]. With the interplay between skills and qualities, it can be difficult to highlight and recognise team qualities within IPE settings [26]. Using a game‐based activity such as ERiB to facilitate these team qualities is a useful tool to distinguish and highlight these attributes in a team environment. ERiB appeared effective in fostering and emphasising these collaborative capability qualities by promoting mutual respect, shared accountability and valuing of diverse strengths. Students demonstrated adaptability, perspective‐taking and psychological safety within heterogeneous teams. These experiences appear to strengthen relational competencies foundational to effective interprofessional and rural health practice. The immersive and interactive nature of ERiB appeared to accommodate diverse abilities and generational learning preferences while strengthening interpersonal competencies crucial to health practice [15]. Collectively, these findings suggest that immersive, team‐based learning may contribute meaningfully to the development of collaborative capability among rural health students.

Peña and Koch [27] emphasise training of humility as critical to fostering respect and openness, promoting collaborative attitudes essential in interprofessional practice. ERiB appeared to cultivate qualities of humility, such as situational humility, by minimising hierarchies and promoting psychological safety. Students demonstrated openness, self‐awareness and respect for others' expertise—attributes essential for effective interprofessional collaboration [27, 28]. These capabilities are particularly relevant within rural health contexts requiring adaptive, team‐based practice. Irving et al. [29] state that poor use of effective communication, flexibility and teamwork were barriers to supporting clinicians and students living and working in rural areas. The luxury of stable staffing with well‐defined tasks and effective routines has been surpassed by a dynamic shift to fluidity to adapt to the evolving nature of health care and patient focus, where a unique set of protocols is often required and professionals may be reluctant to take interpersonal risks [3]. Students demonstrated openness, self‐awareness and respect for others' expertise—attributes essential for effective interprofessional collaboration. Students reported the opinions of members were valued by other team members and that input from other team members was listened to and considered. These capabilities are particularly relevant within rural health contexts requiring adaptive, team‐based practice.

To prepare students to become collaborative practice‐ready health professionals, intrinsic motivation underscores fundamental team values required for team collaboration and interprofessional practice [30]. Motivation is seen to exist along a continuum ranging from amotivation, a negative state lacking drive or interest in completing a task, through to intrinsic motivation, the positive situation where individuals are autonomous, deriving pleasure and interest in completing an activity. Gamification as a pedagogical strategy, engaging learners in problem‐solving tasks and game‐based challenges to achieve specific outcomes, enhances intrinsic motivation [4]. ERiB elicited intrinsically motivated engagement characterised by enjoyment, persistence and shared purpose. Students reported feeling more connected to peers from other disciplines and were better able to appreciate the value of communication, teamwork and mutual problem solving; critical competencies for interprofessional collaboration. Veldkamp et al. [31] strengthen this further by outlining that when interprofessional competences are practiced and developed in an escape room, students reported increased engagement when compared with regular learning activities. Such internally driven motivation in the ERiB context shows students the importance of strengthening collaborative learning in preparing for rural clinical practice.

Collectively, the four themes illustrate the multidimensional impact of the ERiB activity on students' professional development. The integration of affective challenge, shared problem‐solving and psychological safety created conditions that supported autonomy, competence and relatedness, consistent with Self‐Determination Theory. Through navigating uncertainty together in the ERiB activity, students demonstrated interpersonal awareness and adaptive teamwork reflective of traits essential for developing their professional identity.

The role of the facilitator emerged as a pivotal factor in the success of the learning activity. Effective facilitation involved providing timely support, managing group dynamics and ensuring students remained engaged without reducing the challenge of the task. Inadequate facilitation, for example, inappropriate hints or prompts, resulted in disengagement and reduced learning outcomes. The facilitator had to observe students for signs of frustration and be familiar with the game and intervene at the right time, not too soon that there is no conflict but not too late that they disengage. This echoes prior research that highlighted the importance of guided learning and educator adaptability in team‐based learning [32, 33]. To support consistency across diverse rural sites, the project team implemented a comprehensive facilitator guide and standardised debrief script, which proved essential for maintaining quality and achieving learning objectives. This worked well with a large team of clinical educators spread across the UDRH region.

### 4.1 Limitations

ERiBs briefcase‐style design enables easy transport and set up in any location, however, a key consideration was maintaining durability; for example, exposure to extreme heat caused damage to a briefcase, necessitating redesign and better storage protocols.

Each workshop required at least two clinical educators, which could be deemed as resource heavy in terms of facilitator time. The trade‐off was a highly engaging and educational experience that could be deployed even in placements based in remote areas. The consistent positive feedback from students and educators suggests that the investment is justified, particularly given the limited opportunities for high‐quality interprofessional learning in rural settings.

The cost of the briefcases and the time taken to build each case could also be viewed as a limitation. As the briefcases were built by external contractors. Multiple consultations were needed for each briefcase build, to ensure it was themed appropriately to the clinical scenario. The uniqueness, flexibility and transportability of the briefcases as an educational resource provided the justification for the cost and time needed to build them.

## 5 Conclusion

Escape rooms have been shown to provide an enjoyable game‐based environment where interprofessional competencies and teamwork behaviours can be developed. The portability and flexibility of ERiB address a longstanding gap in rural health education by making high‐quality IPE accessible to students in geographically isolated placements.

Effective facilitation and structured debriefing were critical to the success of the activity, helping students translate their experiences into reflective learning. With appropriate support, ERiB offers a sustainable and scalable model for interprofessional education.

As healthcare evolves, immersive, team‐based learning approaches such as ERiB may help prepare students for collaborative practice in the complex contexts of rural health. These findings suggest that non‐clinical, game‐based activities can support the development of interprofessional practice capabilities and professional identity while reducing hierarchical barriers and fostering teamwork.

## Author Contributions

**Lucy Parker:** conceptualization, investigation, writing – original draft, methodology, writing – review and editing, formal analysis, project administration, resources. **Kirsten Middleton:** investigation, writing – original draft, formal analysis, writing – review and editing. **Emma Gordon:** formal analysis, writing – review and editing. **Kate Beyer:** conceptualization, investigation, writing – original draft, methodology, writing – review and editing, formal analysis, project administration, resources. **Flora Rolf:** methodology, formal analysis, writing – review and editing. **Christine O'Connell:** investigation, writing – original draft, writing – review and editing, methodology. **Anthony Fallon:** writing – review and editing. **Dayle Osborn:** writing – review and editing, formal analysis.

## Ethics Statement

This study was approved by the University of Queensland Human Research Ethics Committee (approval number 2021/HE001982). All participants provided informed consent prior to participation and the research was conducted in accordance with the National Statement on Ethical Conduct in Human Research.

## Conflicts of Interest

The authors declare no conflicts of interest.

## Data Availability

The data that support the findings of this study are available from the corresponding author upon reasonable request.

## Appendix A Escape Room in a Briefcase (ERiB) Clinical Scenario and Associated Puzzle Sequence

The following flow chart illustrates the progression of a simulated clinical case and embedded puzzles within the Escape Room in a Briefcase (ERiB) activity.

Rectangular boxes on the left indicate stages in the patient journey and clinical narrative. Boxes on the right illustrate a puzzle component that aligns with the narrative. Arrows demonstrate the sequence of events. Decision points and task‐based activities are embedded as puzzle components, requiring participants to apply interprofessional collaboration to progress. Puzzle outcomes generate codes or prompts that unlock subsequent stages. The scenario integrates clinical, psychosocial, and contextual factors relevant to rural healthcare, culminating in the selection of an appropriate interprofessional management plan.

## Appendix B Focus Group Interview Questions

The following table outlines the prompts and questions asked during the debrief session. Additional questions may have been asked to request explanations or clarify what was discussed.

**Table:**

Questions	Additional prompting questions if required
*The gaming experience*	
Share with us your thoughts about your experience while being involved in the Escape Room in a Briefcase activity	When you were playing the Escape Room in a Briefcase, how did it make you feel? Did you find it enjoyable? Did it create any stress or anxiety for you? Can you remember any points in the game that were particularly memorable? Can you explain why? Do you think it suits some learners better than others?
*Positive change in interprofessional competency domains*	
What IP competencies did you see during the Escape Room in a Briefcase activity?	Can you give us an example of a situation during the Escape Room in a Briefcase activity where you saw something relevant to interprofessional competencies? (Remind participants of the interprofessional competencies to assist) Do you think the Escape Room in a Briefcase activity was a helpful way of learning about or consolidating your knowledge of the interprofessional competencies?
*Teaming skills*	
One of the intentions of the Escape Room in a Briefcase activity is to bring a new team of diverse people together to solve a set of complex problems. Do you think that this activity achieved this objective?	Did you learn about how your other team members interacted with each other? Was there anything new, surprising or unexpected? Did you feel comfortable making suggestions? Did you feel listened to? Were there times during the activity where you tried different approaches to solving a problem? If so, tell us about what happened? Did the Escape Room in a Briefcase activity give you an opportunity to reflect on how diverse teams work together to solve problems? What did you learn? Will this inform your future practice when working within healthcare teams? If so, how will it?
*Processes*	
Did the Escape Room in a Briefcase activity run smoothly? Please comment on the ease or difficulty of the problems you needed to solve Do you have any comments on the way the facilitators conducted the Escape Room in a Briefcase activity?	Are there any ways in which the Escape Room in a Briefcase could be improved, in terms of: ○ The activity itself, or ○ The way the activity was conducted How was the debrief session following the Escape Room in a Briefcase activity? ○ Did you feel comfortable expressing your thoughts and feelings about the activity? ○ Did have sufficient opportunity and time to do so?

## References

[1] World Health Organisation , Framework for Action on Interprofessional Education and Collaborative Practice (World Health Organisation, 2010), https://www.who.int/publications/i/item/framework‐for‐action‐on‐interprofessional‐education‐collaborative‐practice. 21174039

[2] H. Nawaz , A. Edmondson , T. Tzeng , J. Saleh , K. Bozic , and K. Saleh , “Teaming: An Approach to the Growing Complexities in Health Care,” Journal of Bone and Joint Surgery 96 (2014): e184, 10.2106/JBJS.M.01268. 25378517

[3] A. Edmonson , Teaming: How Organizations Learn, Innovate, and Compete in the Knowledge Economy (Jossey‐Bass, 2012).

[4] J. Gomez‐Urquiza , J. Gomez‐Salgado , L. Albendin‐Garcia , M. Correa‐Rodriguez , E. Gonzalez‐Jimenez , and G. Canadas‐De la Fuente , “The Impact on Nursing Student's Opinions and Motivation of Using a ‘Nursing Escape Room’ as a Teaching Game: A Descriptive Study,” Nurse Education Today 72 (2019): 73–76. 30453202 10.1016/j.nedt.2018.10.018

[5] Y. G. Kwon , M. Namgung , S. H. Park , et al., “Impact of a Game‐Based Interprofessional Education Program on Medical Students' Perceptions: A Text Network Analysis Using Essays,” BMC Medical Education 24 (2024): 898, 10.1186/s12909-024-05893-2. 39164644 PMC11334522

[6] L. Moore and N. Campbell , “Effectiveness of an Escape Room for Undergraduate Interprofessional Learning: A Mixed Methods Single Group Pre‐Post Evaluation,” BMC Medical Education 21, no. 1 (2021): 220, 10.1186/s12909-021-02666-z. 33879150 PMC8056636

[7] J. W. Creswell and C. N. Poth , Qualitative Inquiry & Research Design: Choosing Among Five Approaches, Fourth ed. (SAGE, 2019).

[8] V. Braun and V. Clarke , “Using Thematic Analysis in Psychology,” Qualitative Research in Psychology 3, no. 2 (2006): 77–101.

[9] Australian Government , “Modified Monash Model,” (2025), https://www.health.gov.au/topics/rural‐health‐workforce/classifications/mmm.

[10] J. M. Lodge , G. Kennedy , L. Lockyer , A. Arguel , and M. Pachman , “Understanding Difficulties and Resulting Confusion in Learning: An Integrative Review,” Frontiers in Education 3 (2018): 49.

[11] A. Amsel , Frustration Theory: An Analysis of Dispositional Learning and Memory (Cambridge University Press, 1992).

[12] S. D'Mello , B. Lehman , R. Pekrun , and A. Graesser , “Confusion Can Be Beneficial for Learning,” Learning and Instruction 29 (2014): 153–170.

[13] A. Davison , P. Willis , and T. Leonard , “Myth in the Practice of Reason: The Production of Education and Productive Confusion,” in Pedagogies of the Imagination (Springer Netherlands, 2008), 53–63, 10.1007/978-1-4020-8350-1_4.

[14] D. Burks and A. Kobus , “The Legacy of Altruism in Health Care: The Promotion of Empathy, Prosociality and Humanism,” Medical Education 46 (2012): 317–325. 22324531 10.1111/j.1365-2923.2011.04159.x

[15] N. Rowley and S. Lucas , “Evaluating Student Nurses Satisfaction With Educational Escape Rooms as a Pedagogical Approach to Teaching Professional Nursing Values,” BMC Nursing 24 (2025): 1300, 10.1186/s12912-025-03912-1. 41121180 PMC12542332

[16] R. M. Ryan and E. L. Deci , “Self‐Determination Theory,” in Encyclopedia of Quality of Life and Well‐Being Research, ed. F. Maggino (Springer, 2022), 10.1007/978-3-319-69909-7_2630-2.

[17] Y. Zhu , R. A. Kusurkar , D. Dolmans , et al., “Evaluating and Understanding Undergraduate Students' Basic Psychological Needs Satisfaction and Motivation Across Different Learning Environments: A Mixed‐Methods Study,” Learning Environments Research 29 (2026): 9, 10.1007/s10984-025-09558-9.

[18] A. Manzano‐León , J. M. Rodríguez‐Ferrer , J. M. Aguilar‐Parra , et al., “Escape Rooms as a Learning Strategy for Special Education Master's Degree Students,” International Journal of Environmental Research and Public Health 18, no. 14 (2021): 7304, 10.3390/ijerph18147304. 34299753 PMC8305373

[19] B. Michalec , D. Papanagnou , L. Raj , et al., “Exploring the Presence and Roles of Humility When Experiencing Situations of Uncertainty,” AEM Education and Training 9, no. 1 (2025): e11055, 10.1002/aet2.11055. 39846033 PMC11745895

[20] L. Gleeson , G. L. O'Brien , D. O'Mahony , and S. Byrne , “Interprofessional Communication in the Hospital Setting: A Systematic Review of the Qualitative Literature,” Journal of Interprofessional Care 37, no. 2 (2023): 203–213, 10.1080/13561820.2022.2028746. 35109753

[21] L. T. Hong , P. Gavaza , J. Koch , and I. de la Peña , “An Explorative Study on Intellectual Humility Differences Among Health Professional Students: Implications for Interprofessional Education and Collaboration,” Journal of Interprofessional Education and Practice 33 (2023): 100674, 10.1016/j.xjep.2023.100674.

[22] M. Vansteenkiste , R. M. Ryan , and B. Soenens , “Basic Psychological Need Theory: Advancements, Critical Themes, and Future Directions,” Motivation and Emotion 44, no. 1 (2020): 1–31, 10.1007/s11031-019-09818-1.

[23] L. M. Jeno , A. Raaheim , S. M. Kristensen , et al., “The Relative Effect of Team‐Based Learning on Motivation and Learning: A Self‐Determination Theory Perspective,” CBE Life Sciences Education 16, no. 4 (2017): ar59, 10.1187/cbe.17-03-0055. 29146665 PMC5749961

[24] L. Moore and N. Campbell , “Short Report: Novel Interprofessional Learning for Healthcare Students: An Escape Room Pilot,” Focus on Health Professional Education: A Multi‐Professional Journal 20, no. 1 (2019): 1–7.

[25] E. McKinlay , T. Gladman , M. Burrow , and S. Pullon , “Health Professional Students' Emotional Responses to Effective and Ineffective Teamwork,” Focus on Health Professional Education 25, no. 3 (2024): 103–120, 10.11157/fohpe.v25i3.692.

[26] I. Paton , N. Patton , and A. Croker , “Allied Health Collaborative Practice Capability: A Coalescence of Capabilities,” Journal of Allied Health 53, no. 1 (2024): 45–50. 38430496

[27] I. Peña and J. Koch , “Teaching Intellectual Humility Is Essential in Preparing Collaborative Future Pharmacists,” American Journal of Pharmaceutical Education 85, no. 10 (2021): 8444, 10.5688/ajpe8444. 34965915 PMC8715963

[28] B. Michalec , A. Xyrichis , and C. Arenson , “‘Professional Humility’: Introducing a New Framework to Advance Interprofessionalism,” Journal of Interprofessional Care 38, no. 4 (2024): 1–6, 10.1080/13561820.2024.2326974. 38501441

[29] M. Irving , S. Short , K. Gwynne , M. Tennant , and A. Blinkhorn , “‘I Miss My Family, It's Been a While…’ A Qualitative Study of Clinicians Who Live and Work in Rural/Remote Australian Aboriginal Communities,” Australian Journal of Rural Health 25, no. 5 (2017): 260–267. 28008684 10.1111/ajr.12343

[30] F. A. Ganotice , C. S. Chan , E. W. Y. Chan , et al., “Autonomous Motivation Predicts Students' Engagement and Disaffection in Interprofessional Education: Scale Adaptation and Application,” Nurse Education Today 119 (2022): 105549, 10.1016/j.nedt.2022.105549. 36182789

[31] A. Veldkamp , L. van de Grint , M. C. P. J. Knippels , and W. R. van Joolingen , “Escape Education: A Systematic Review on Escape Rooms in Education,” Educational Research Review 31 (2020): 100364, 10.1016/j.edurev.2020.100364.

[32] J. Reuter , D. M. Ferreira , M. Amorim , M. Madaleno , and C. Veloso , “Educators as Facilitators of Game‐Based Learning,” in Handbook of Research on Acquiring 21st Century Literacy Skills Through Game‐Based Learning (IGI Global, 2022).

[33] J. W. Tan , G. Tan , X. Lian , et al., “Impact of Facilitation on Cognitive Flow in a Novel Diabetes Management Rehearsal Game for Health Professions Education: Mixed Methods, Open‐Label, Superiority Randomized Controlled Trial,” JMIR Serious Games 12 (2024): e54703, 10.2196/54703. 38900700 PMC11292155

